# Tri-Tek (Petroleum Horticultural Oil) and *Beauveria bassiana*: Use in Eradication Strategies for *Bemisia tabaci* Mediterranean Species in UK Glasshouses

**DOI:** 10.3390/insects6010133

**Published:** 2015-02-12

**Authors:** Andrew G. S. Cuthbertson, Debbie A. Collins

**Affiliations:** The Food and Environment Research Agency, Sand Hutton, York YO41 1LZ, UK; E-Mail: debbie.collins@fera.gsi.gov.uk

**Keywords:** *Bemisia tabaci*, chemical, entomopathogenic fungi, eradication

## Abstract

The sweetpotato whitefly *Bemisia tabaci* (Gennadius) (Hemiptera: Aleyrodidae) is a pest of global importance on both outdoor and glasshouse crops. To date, *B. tabaci* has not become established in the UK. The UK holds Protected Zone status against this pest and, as a result, *B. tabaci* entering on plant material is subjected to a policy of eradication. Mediterranean species is now the most prevalent *Bemisia* species entering the UK. Increasing neonicotinoid resistance is becoming increasingly widespread and problematic with this species. As a result, this continues to pose problems for eradication strategies. The current study investigates the efficacy of Tri-Tek (a petroleum horticultural oil awaiting UK registration) and the fungus *Beauveria bassiana* to act as control agents against Mediterranean species in UK glasshouses. Tri-Tek provided 100% egg mortality compared to 74% for *B. bassiana.* When tested against second instar larvae, mortalities of 69% and 65% respectively were achieved. Both products can be successfully “tank-mixed”. A tank-mix application provided 95.5% mortality of second instar larvae under glasshouse conditions. The potential integration of both products into current *Bemisia* eradication strategies in UK glasshouses is discussed.

## 1. Introduction

The sweetpotato whitefly, *Bemisia tabaci* (Gennadius) (Hemiptera: Aleyrodidae) is regarded as a major pest worldwide [1,2] which causes direct yield loss and economic damage in many commercially important crop species [3,4,5]. Direct damage is caused by its feeding activity and indirectly due to contamination of leaves with honey dew on which black mould develops and intercepts light, thereby reducing photosynthesis [2].

The pest status of *B. tabaci* insects is complicated by the recognition of 11 well-defined genetic groups and at least 24 morphocryptic species which are morphologically identical but distinguishable at the molecular level [6,7]. The damaging Middle East Asia Minor 1 (MEAM1) species (formerly known as B biotype) is of specific economic concern because it is an effective vector of over 111 viruses from several families, particularly geminiviruses [8], many of which are not present in the UK [9,10].

*Bemisia tabaci* continues to be regularly intercepted in the UK on imported plant material, mainly associated with poinsettia cuttings [10], where it is subject to statutory controls aimed at eradication [10,11]. In recent years there has been a shift from MEAM1 to Mediterranean species (formerly known as Q biotype) entering the UK [12]. This has led to increased problems in regards to eradication strategies currently employed due to increased chemical resistance shown by *B. tabaci* Mediterranean. Control of *B. tabaci* is difficult because of morphological and autecological characteristics (waxy substances on the cuticle, colonisation of the underside of leaves and the rapid development of very dense populations) as well as the ability to develop resistance to a number of pesticides [4,13,14,15,16,17]. Therefore, an integrated programme combining biological, chemical and physical controls is needed if complete eradication is to be reliably achieved. Until recently, in the UK, *B. tabaci* outbreaks were often treated using the chemicals buprofezin, nicotine, imidacloprid or teflubenzuron [14,18,19,20]. However, a recent study by Cuthbertson *et al.* [11] has shown excellent potential of several products (including entomopathogenic fungi) to eradicate *B. tabaci* Mediterranean under controlled laboratory conditions. However, some products (Tri-Tek (petroleum horticultural oil) and *Beauveria bassiana*) have not been tested under UK glasshouse conditions.

The development of control strategies for non-indigenous insects is limited by UK legislation which precludes the intentional release of quarantine pests into ordinary experimental glasshouses [21]. In the current study, the potential of products (Tri-Tek and *Beauveria bassiana*) shown to have a high efficacy against *B. tabaci* Mediterranean species under laboratory conditions [11] were used to test their efficacy against the whitefly under glasshouse conditions using designated quarantine glasshouse cubicles at The Food and Environment Research Agency, York.

## 2. Experimental Section

### 2.1. Products and Insect Cultures

*Bemisia tabaci* Mediterranean species [12] was cultured under quarantine conditions in perspex cages (60 cm × 60 cm × 80 cm) on poinsettia (*Euphorbia pulcherrima* Wild. ex Klotzsch c.v Lilo Pink (Euphorbiaceae)) plants as hosts at 23 ± 1 °C with a 16:8 h Light:Dark (L:D) regime and an artificial dawn and dusk [22]. The entomopathogenic fungus *Beauveria bassiana* was supplied as Naturalis from Intrachem, Italy. Tri-Tek, a refined petroleum horticultural oil and physically acting product (awaiting UK registration) was supplied by Brandt Ltd. (Springfield, IL, USA).

### 2.2. Efficacy against *Bemisia tabaci* Mediterranean Eggs

Poinsettia plants (an important UK horticultural commodity) were chosen for testing the efficacy of the two products under glasshouse conditions. Sixteen plants (eight treated and eight control) were infested and incubated as outlined in Cuthbertson *et al.* [11]. Briefly, four clip cages, modelled on those described by MacGillivray and Anderson [23], were positioned randomly on individual leaves (one cage per leaf) of each of 16 plants (eight treated and eight control). Two male and five female *B. tabaci* were added to each cage and incubated for 48 h at 25 ± 1 °C, 65% relative humidity (r.h.) and 16:8 h Light:Dark (L:D) to allow egg laying, after which adults were removed and infested leaves labelled. After egg laying had occurred (approximately 20–30 eggs produced) and the adults (along with the clip cages) had been removed, the plants were transferred in sealed cooler boxes to the designated glasshouse cubicle. The plants were arranged randomly throughout the cubicle. The conditions of the cubicle were set for 21°C (average UK glasshouse temperature [24]). One hour before spraying occurred, the cubicle and plants were soaked with water in order to raise the humidity (>85%) [21]. The plants were then sprayed with *B. bassiana* at a rate of 1 × 10^7^ conidia/ml using a hand held Hozelock^®^ Polyspray 2 hand held sprayer with a cone nozzle (Hozelock Ltd., Aylesbury, UK), during late evening on a dull, overcast day. Following spraying, plants remained within the cubicle for a further seven days before being taken back to the quarantine laboratory in sealed cooler boxes for analysis of treatment effect. The procedure was repeated for testing efficacy of Tri-Tek (as a 2% solution) and also a Tri-Tek and *B. bassiana* tank-mix (2% Tri-Tek and 1 × 10^7^ *B. bassiana* conidia/mL) against *B. tabaci* eggs. Previous work has shown that Tri-Tek and *B. bassiana* can be successfully tank-mixed [11]. Equal numbers of plants were sprayed with water and acted as controls.

### 2.3. Efficacy against *Bemisia tabaci* Mediterranean 2nd Instars

Following the method above, when infested plants (16 in total; eight treated: eight control) were transferred to the glasshouse cubicle they were further incubated at 25 °C for another 12 days to allow eggs to hatch and larvae to reach the second larval instar [25,26,27], the stage most susceptible to fungus infection [28]. The temperature was then lowered to 21 °C and the cubicle again sprayed with water to raise the humidity to >85%. Following spraying the plants with *B. bassiana* they remained within the cubicle for a further seven days before being taken back to the quarantine laboratory in sealed cooler boxes for analysis of treatment effect. The procedure was repeated for testing efficacy of Tri-Tek and a Tri-Tek and *B. bassiana* tank-mix against *B. tabaci* 2nd instars. The products were applied at the same rates as outlined above.

### 2.4. Analysis of Data

In all trials whiteflies were recorded as dead or alive. Assessments were made using a dissecting microscope. Following Tri-Tek treatment numbers of live and dead *Bemisia* larvae were recorded after two days. In the case of the fungal treatments and all the *Bemisia* egg trials, plants were incubated for seven days to allow the fungus to germinate and eggs to potentially hatch. Treated eggs were noted as live (hatched larvae) or dead (unhatched). The data underwent non-parametric method testing (Kruskal-Wallis rank sum test and Wilcoxon test) to determine the effect of treatments where necessary.

## 3. Results and Discussion

*Bemisia tabaci* Mediterranean is the dominant whitefly entering the UK on imported plant material [12]. This whitefly is widely considered to evolve stable resistance to the neonicotinoid insecticides commonly used for *Bemisia* control more rapidly than MEAM1 [29]. Hence, Mediterranean neonicotinoid resistance is becoming increasingly widespread and problematic, with numerous cases being reported worldwide [30,31,32,33]. As a result, this continues to pose problems for eradication programmes in the UK and the maintenance of “Protected Zone” status where eradication of *B. tabaci* has traditionally centered on the use of neonicotinoids [9]. For eradication programmes to be successful a given treatment must provide total mortality of the pest.

**Figure 1:**
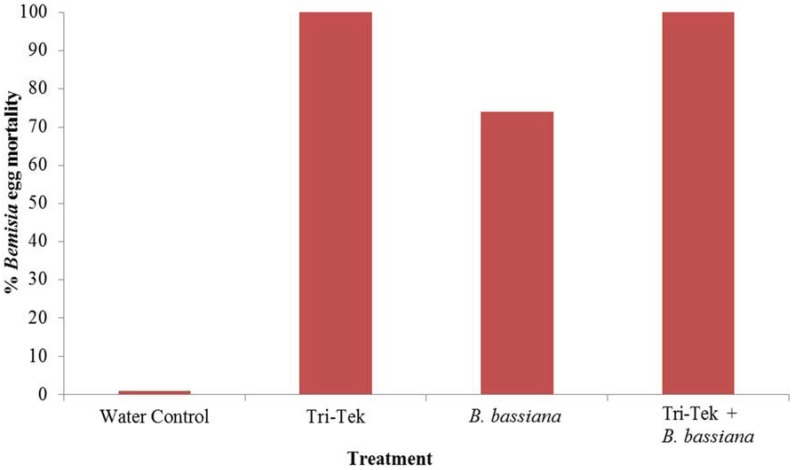
Efficacy of Tri-Tek and *Beauveria bassiana* and a tank-mix of both products against *Bemisia tabaci* Mediterranean eggs under glasshouse conditions. Mortality assessed following seven days after treatments.

**Figure 2:**
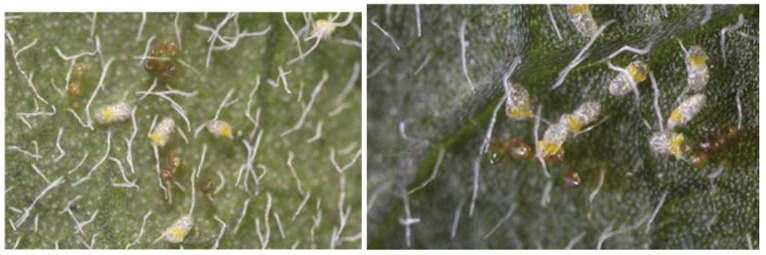
Impact of Tri-Tek on *Bemisia tabaci* Mediterranean eggs. Some eggs hatched but larvae were detached from leaf surface and subsequently died (Photos: Andrew G. S. Cuthbertson^©^).

Tri-Tek proved excellent against *B. tabaci* Mediterranean eggs with 100% mortality being achieved (Figure 1), significantly better than the water control (*p* < 0.001). *Beauveria bassiana* provided 74% egg mortality, significantly less than the Tri-Tek treatment (*p* < 0.005). Tank-mixing the two products also produced 100% mortality. Interestingly, some eggs did hatch in the Tri-Tek treatments and larvae appeared but these did not develop. They appeared dehydrated and detached from the leaf surface; obviously they were not feeding (Figure 2; [34]). Tri-Tek must have been offering some repellent effect or perhaps the young larvae could not penetrate through the residue layer produced by the product in order to feed on the leaf.

**Figure 3:**
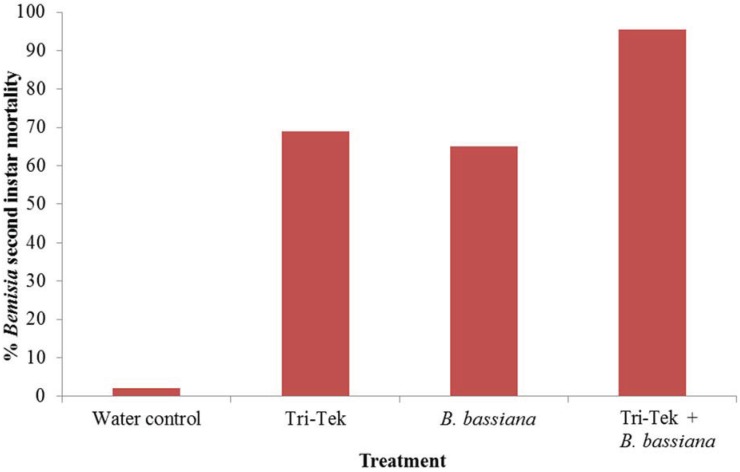
Efficacy of products against *Bemisia tabaci* Mediterranean second instar larvae. Mortality recorded after two days following treatment with Tri-Tek and after seven days following *Beauveria bassiana* treatments.

Against 2nd instar *B. tabaci* Mediterranean larvae Tri-Tek and *B. bassiana* provided 69% and 65% mortality respectively, both significantly higher than the control (*p* < 0.005). Tank-mixing the products increased mortality to 95.5% (Figure 3); displaying some evidence of a synergistic effect. This may be due to the oil protecting the fungal conidia in some way; perhaps by retaining moisture.

Currently, within the UK, there are protocols that effectively give eradication of *Bemisia* *tabaci* Mediterranean species on infected plant produce [11]. Both MEAM1 and Mediterranean can successfully be eradicated through sequential treatments of various products [11]. This study has proven that both Tri-Tek and *B. bassiana* offer potential to be also used as control agents against *B. tabaci* Mediterranean in UK glasshouses. In the current study, as also noted by Cuthbertson *et al* [11], no phytotoxic effects were noted following the application of Tri-Tek to the poinsettia plants. McKenzie *et al.* [35] demonstrated that the use of various oils did cause phytotoxic effects on various varieties of poinsettia and recommended that they should be used early in chemical rotation programmes. Further repeated applications of Tri-Tek at various concentrations, and on different plant foliage, would give more valuable information in regards to its suitability for incorporation into eradication strategies. Najar-Rodriguez *et al.* [36] reports that petroleum spray oils not only act by suffocation but also affect nerve activity of insects and so could be integrated into resistance management strategies for synthetic insecticides that target the central nervous system of insects. The oil could be applied in conjunction with such insecticides to kill resistant individuals that survive the insecticide application. It is unknown if Tri-Tek acts is such a manner.

Upgrading of eradication strategies is essential in order to keep one step ahead of the insect pest in question. An ever increasing scenario facing the UK is that other cryptic species of *Bemisia* are now also being intercepted on produce [37]. These will no doubt cause problems in regards to ensuring successful eradication in the future.

## 4. Conclusions

*Bemisia tabaci* remains a severe threat to UK horticulture [10]. The continuing interception of insecticide resistant *B. tabaci* Mediterranean species poses a real challenge in regards to maintaining the UK’s Protective Zone status. *Beauveria bassiana* has shown potential to be included in eradication strategies against *B. tabaci*. Also, the potential arrival of Tri-Tek onto the UK market provides another tool to be included within current eradication strategies; so helping to ensure the UK remains *Bemisia* free.
